# Synergistic tomographic image reconstruction: part 2

**DOI:** 10.1098/rsta.2021.0111

**Published:** 2021-08-23

**Authors:** Charalampos Tsoumpas, Jakob Sauer Jørgensen, Christoph Kolbitsch, Kris Thielemans

**Affiliations:** ^1^ Biomedical Imaging Science Department, University of Leeds, West Yorkshire, UK; ^2^ Biomedical Engineering and Imaging Institute, Icahn School of Medicine at Mount Sinai, New York, NY, USA; ^3^ Invicro, London, UK; ^4^ Department of Applied Mathematics and Computer Science, Technical University of Denmark, Kongens Lyngby, Denmark; ^5^ Department of Mathematics, The University of Manchester, Manchester, UK; ^6^ Physikalisch-Technische Bundesanstalt, Braunschweig and Berlin, Germany; ^7^ School of Biomedical Engineering and Imaging Sciences, King’s College London, London, UK; ^8^ Institute of Nuclear Medicine, University College London, London, UK; ^9^ Centre for Medical Image Computing, University College London, London, UK

**Keywords:** positron emission tomography, X-ray computed tomography, magnetic resonance imaging, diffuse optical tomography, tomography, open-source software

## Abstract

This special issue is the second part of a themed issue that focuses on synergistic tomographic image reconstruction and includes a range of contributions in multiple disciplines and application areas. The primary subject of study lies within inverse problems which are tackled with various methods including statistical and computational approaches. This volume covers algorithms and methods for a wide range of imaging techniques such as spectral X-ray computed tomography (CT), positron emission tomography combined with CT or magnetic resonance imaging, bioluminescence imaging and fluorescence-mediated imaging as well as diffuse optical tomography combined with ultrasound. Some of the articles demonstrate their utility on real-world challenges, either medical applications (e.g. motion compensation for imaging patients) or applications in material sciences (e.g. material decomposition and characterization). One of the desired outcomes of the special issues is to bring together different scientific communities which do not usually interact as they do not share the same platforms such as journals and conferences.

This article is part of the theme issue ‘Synergistic tomographic image reconstruction: part 2’.

## 1. Introduction

Image reconstruction describes the process from acquired data to the corresponding image information. Often research in this area is guided by the individual modalities (e.g. magnetic resonance imaging (MRI), X-ray computed tomography (CT) and positron emission tomography (PET) for medical imaging) or by different fields (e.g. tomographic imaging in medicine, biology or industry). Although each modality has its own unique challenges, the underlying image reconstruction problems also have a lot in common and the mathematical, computational and software aspects are not necessarily so different.

Each of these areas uses innovative solutions to optimize the reconstructed images. However, there is a lot of synergy between different fields and modalities. The special issue focuses on ideas or methodologies that could be translated across domain areas and hence benefit many different applications. Thus, we hope that the special issue will help accelerate future research across different scientific fields. The common element of the research and review articles is the synergy of different measurements to provide substantially improved information on the unknown quantity/quantities being reconstructed. The approach to synthesizing and combining information intelligently is of great relevance in various research domains beyond the scope of this special issue and we envisage that techniques and ideas presented in this issue may attract interest in completely different application domains.

In order to bring together scientists and academics across different research domains, we organized in November 2019 a symposium on Synergistic Image Reconstruction (http://www.ccpsynerbi.ac.uk/symposium2019) and this special issue includes work presented at this event. The meeting was sponsored by two EPSRC-sponsored networks: the collaborative computational project (CCP) in synergistic PET-MR image reconstruction (CCP-PET-MR, http://www.ccppetmr.ac.uk) and the collaborative computational project in tomographic imaging (CCPi, https://www.ccpi.ac.uk/). Both these networks, which will stay active at least until 2025, with the former expanded to synergistic reconstruction in biomedical imaging (CCP SyneRBI, https://www.ccpsynerbi.ac.uk/), welcome national and international collaborations to better support their scientific communities.

The special issue entitled ‘Synergistic Tomographic Reconstruction‘ is separated in two different parts. The first issue (Part 1) included three review articles and five research investigations [1]. This is the second part of the issue, and focuses on novel concepts as well as methods and software tools that can be useful for multi-spectral and multi-modality imaging such as spectral X-ray CT, MRI, PET-MRI, PET/CT, bioluminescence imaging and fluorescence-mediated imaging as well as diffuse optical tomography with ultrasound. Many of these techniques have been developed only recently and several potential synergies still need to be explored [2].

## 2. Contributions in the second issue

The issue commences with a review article by Polycarpou *et al.* [3] which summarizes various recently proposed motion compensation methods in PET-MRI. Physiological motion of the subject during scanning can affect the diagnostic value of PET-MRI substantially. The synergistic nature of integrated PET-MRI scanners can help combine information and potentially correct for motion artefacts in the images. Synergy can be achieved on various levels including motion tracking, estimation and correction. The review presents successful examples and discusses software and data available to the scientific community for validation and further use in clinical and preclinical scanners.

The next contribution—in two parts—presents the Core Imaging Library (CIL), a new open-source Python framework for processing and reconstruction of tomographic imaging data. Both articles provide complete open-access code and data to fully reproduce results and figures.

Part I by Jørgensen *et al.* [4] gives an overview of CIL and its capabilities to handle both conventional but also challenging datasets such as those with high noise, incomplete data, non-standard scan geometries or obtained with new modalities such as dynamic and spectral CT. A modular object-oriented design and a flexible convex (smooth and non-smooth) optimization framework enable users to experiment with existing reconstruction algorithms or easily implement new ones. The capabilities are demonstrated in four case studies covering synchrotron parallel-beam X-ray CT, laboratory cone-beam laminography, golden-angle neutron tomography and positron emission tomography.

**Figure 1.**
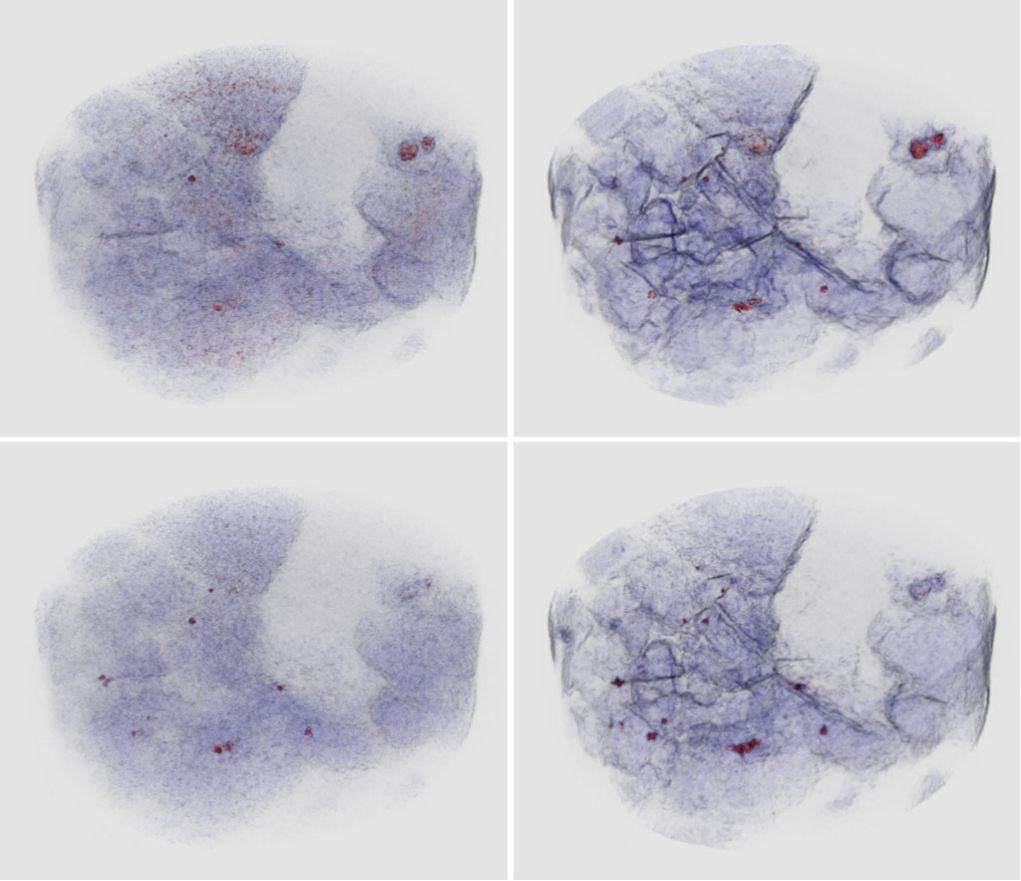
Example from [5] of hyperspectral X-ray tomographic imaging in three dimensions of gold and other mineral deposits using synergistic reconstruction methods. Localizing and distinguishing inclusions with similar densities is not possible using conventional X-ray CT. New energy-sensitive X-ray detectors can, in principle, distinguish inclusions through K-edge absorption profiles which act as elemental fingerprints. However, the hyperspectral data is very noisy, causing simple reconstruction of energy channels independently to fail: channelwise SIRT reconstruction (left column) of energy channel below (top row) and above (bottom row) gold and galena K-edges are noisy and inclusions poorly defined. Using a synergistic reconstruction method described in [5] employing both spectral and spatial regularization (right column, same energy channels as before in top and bottom rows) the imaging of mineral phases and small inclusions is strongly improved. Inclusions can be distinguished due to different responses at different energies. For example, unlike the many smaller inclusions (gold and galena) the two large inclusions in the top right only show a high response at the lower energy, which helps to distinguish these as chalcopyrite. Image credit: Laura Murgatroyd, STFC, UKRI.

Part II by Papoutsellis *et al.* [5] focuses on the capabilities of CIL for multi-channel cases such as dynamic (time-resolved) and spectral (energy-resolved) tomography. CIL natively supports multi-channel data and provides a number of dedicated regularization methods, such as spatial, spectral and spatio-spectral variants of Total Variation (TV). CIL handles general linear inverse problems, as demonstrated by the first case study of colour denoising and inpainting. The second case study covers fast dynamic CT and compares a range of regularized reconstruction methods including directional TV in terms of compensating for highly undersampled data and exploiting the dynamic dimension. The third and final case study considers K-edge imaging in hyperspectral tomography. The use of CIL algorithms and building blocks demonstrate how cross-channel regularization can produce substantial improvements in reconstruction quality, and the use of a stochastic algorithm to accelerate computations is further discussed (figure 1).

Motion-correction for PET-MRI is the subject of the next paper by Brown *et al.* [6]. It describes recent additions to the open-source Synergistic Image Reconstruction Framework (SIRF) [7], enabling researchers to develop and evaluate new methods of motion correction and image reconstruction for either PET, MRI or (synergistic) PET-MRI. The article describes the basic functionality and illustrates the software in the context of respiratory motion. In the example, motion estimation is performed on cardiac MRI data. This is then followed by motion-compensated image reconstruction for both MRI and PET, combining functionality of SIRF and CIL to obtain fully corrected PET-MRI images that are spatially matched.

A final software-related contribution by Peter [8] describes a new framework for multi-modality simulation and reconstruction called Musiré. This provides a *bash* script that allows easy simulation for CT, MR, PET but also bioluminescence imaging (BLI) and fluorescence-mediated imaging (FMI), starting from a single object description. The tool is an interface to several standard open-source packages and handles data format conversions. Although currently, all reconstructions are single-modality, the package simplifies data generation for studying synergistic reconstruction methods.

Li *et al.* [9] describe the development of a promising technique that exploits PET/CT data to perform material decomposition (in two components) without the need for dual-energy X-ray CT, as attenuation for high-energy (511 keV) is estimated from time-of-flight PET data [10]. The paper describes a novel method to reconstruct both activity and high energy attenuation from the PET data by using the X-ray CT for guided reconstruction. The method uses a kernel reconstruction with kernels estimated by an autoencoder convolutional neural network (CNN) with the X-ray CT image as input. The article provides proof-of-concept results in a simulation context. Open-source software implementing the method is also made available.

The article by Cueva *et al.* [11] proposes a new variant of directional TV regularization for spectral CT. Specifically, a spectral CT setup with three energy channels is considered and the three energy channel datasets are initially summed and reconstructed using TV-regularization to obtain a side information image. Each energy channel is then reconstructed separately using directional TV regularization with the side information as a reference from which to propagate edge information. Forward–backward splitting and linearized Bregman iterations are compared for solving the resulting optimization problem. Experiments with simulated and real data demonstrate that the proposed method compares favourably with TV-regularization.

Di Sciacca *et al.* [12] present a method for Diffuse Optical Tomographic (DOT) reconstruction using concurrent ultrasound information as guidance. The inverse problem in DOT has a nonlinear forward model and is highly ill-posed. The paper suggests a practical way to guide the three-dimensional DOT reconstruction using a structural prior from a two-dimensional ultrasound acquisition which is segmented and extended to three-dimensional. The paper compares the proposed approach with non-guided Tikhonov regularization on simulated data using the geometry of the proposed system [13]. Open-source software and datasets are made available.

## 3. Conclusion

The contributions in this second part of the special issue provide an overview of the different scientific fields as well as several software-oriented papers. The latter describe open-source software packages enabling researchers to explore synergistic image reconstruction methods. Where possible, authors have ensured that part of their data has been made publicly available via open data platforms (e.g. Zenodo). We believe that open datasets play an important role in accelerating research outcomes and are equally important to open source code. One of the issues we have noted is the lack of access to clinical datasets primarily due to ethical considerations. Another challenge is that extracting raw data (rather than images) is much harder and usually requires the support by the scanner manufacturer. Having a common protocol or file format for making raw data accessible would strongly help the scientific community, especially scientists from less economically advanced countries who otherwise cannot effectively contribute their scientific ideas to this field. It is our hope that the open-access software described in this issue points towards a more inclusive and open-access research environment to engage more scientists in state-of-the-art research projects, potentially supported by strong international collaborations.

## Data accessibility

This article has no additional data.
